# Smoking Obstructive Sleep Apnea: Arguments for a Distinctive Phenotype and a Personalized Intervention

**DOI:** 10.3390/jpm12020293

**Published:** 2022-02-16

**Authors:** Marina Ruxandra Oțelea, Mihaela Trenchea, Agripina Rașcu, Sabina Antoniu, Corina Zugravu, Ștefan Busnatu, Anca Angela Simionescu, Oana Cristina Arghir

**Affiliations:** 1 Clinical Department 5, “Carol Davila” University of Medicine and Pharmacy, 050474 Bucharest, Romania; marina.otelea@umfcd.ro (M.R.O.); agripina.rascu@umfcd.ro (A.R.); 2 Clinical Department II, Faculty of Medicine, Ovidius University of Constanta, 900527 Constanţa, Romania; mtrenchea79@gmail.com (M.T.); arghir_oana@yahoo.com (O.C.A.); 3 Colentina Clinical Hospital, 020125 Bucharest, Romania; 4 Clinical Department II, “Grigore T. Popa” University of Medicine and Pharmacy, 700115 Iasi, Romania; sabinaantoniu@yahoo.com; 5 Department of Fundamental Sciences, “Carol Davila” University of Medicine and Pharmacy, 050474 Bucharest, Romania; corina.zugravu@umfcd.ro; 6 National Institute of Public Health, 050463 Bucharest, Romania; 7 Clinical Department 4, “Carol Davila” University of Medicine and Pharmacy, 050474 Bucharest, Romania; stefan.busnatu@umfcd.ro; 8 “Bagdasar Arseni” Clinical Emergency Hospital, 041915 Bucharest, Romania; 9 Clinical Department 13, “Carol Davila” University of Medicine and Pharmacy, 050474 Bucharest, Romania; 10 Filantropia Clinical Hospital, 011132 Bucharest, Romania

**Keywords:** apnea–hypopnea index, obstructive sleep apnea, smoking, phenotype, obesity

## Abstract

Background: This is the first study that aims to define smoking, with obstructive sleep apnea (OSA), as a phenotype (SOSA). Moreover, we wanted to demonstrate the deleterious effects of the continuation of smoking on OSA. Methods: The cross-sectional study highlighted four dimensions of SOSA: the demographic and anthropometric features, the symptoms, the comorbidities, and the sleep study parameters. This study compared these characteristics between current smokers (CS), those who have never smoked (NS), and ex-smokers (ES) with OSA. Results: More men (83.95% in CS, versus 66.67% in NS) and an earlier onset of OSA (average age = 50.05 in CS, versus 52.26 in NS, *p* = 0.04) were recorded among CS. The distinguishing symptom of CS was daytime sleepiness, with an Epworth score that was significantly higher than in NS. Chronic obstructive pulmonary disease (COPD) was significantly more prevalent in CS (38.27%) than in NS (1.51%) (*p* < 0.001). The severity of OSA, consisting of a higher apnea-hypopnea index, a higher oxygen desaturation index, and a longer time spent below 90% oxygen saturation during sleep was significantly influenced by smoking. Conclusions: The SOSA phenotype includes younger male patients with a higher waist circumference, suggesting central obesity. They have a higher prevalence of COPD and a greater severity of OSA, in correlation with the number of pack-years of smoking.

## 1. Introduction

Smoking exerts a large spectrum of negative effects on the respiratory system. Inflammation, oxidative stress, altered immunity, dysbiosis, and carcinogenesis are the most widely recognized consequences of smoking exposure [1,2,3]. Extensive literature is dedicated to these negative effects. Despite the high prevalence of smoking, its impact on the incidence and severity of obstructive sleep apnea (OSA) is not sufficiently documented, and the existing data are inconsistent [4,5].

Smoking has the capacity to initiate or enhance a variety of aggravating pathogenic mechanisms in the evolution of OSA. Smoking exerts a direct inflammatory effect on the upper respiratory airways and aggravates OSA indirectly, causing bronchitis or emphysema, which further restricts the respiratory flow. The comorbidities associated with smoking, mainly COPD and cardiovascular disease, contribute to the impairment of oxygenation and a higher frequency of the apneas and hypopneas during sleep. Smoking cessation interventions have the potential to correct both abnormalities [6].

In this study, we aimed to identify the main features of OSA in smokers in order to characterize the smoking OSA (SOSA) phenotype.

## 2. Materials and Methods

### 2.1. Study Design and Participants

This was a cross-sectional study which included adult patients referred to the Pulmonary Disease University Hospital, Constanţa, Romania, for sleep-related respiratory disorders over a three-year period. The study group included patients who were diagnosed with OSA, following the recommendation stated in the consensus of the American Sleep Academy [7]. This consensus underlines that a comprehensive sleep evaluation, in conjunction with the results of the nocturnal respiratory polygraphy (NRP), could be used for an obstructive sleep apnea diagnosis. The pulmonologist, which initially evaluated the patients with sleep-related respiratory disorders, performed a detailed anamnesis and clinical examination, and referred patients, whenever necessary, to other specialist consultations (e.g., cardiology, ENT, endocrinology, neurology), and/or to the necessary tests to evaluate respiratory disorders and comorbidities. The diagnosis of OSA in the study group was finally based on the relevant symptoms, such as snoring with apnea, excessive diurnal sleepiness (EDS) evaluated by Epworth Sleepiness Scale, and an apnea–hypopnea index (AHI) of ≥5, recorded by NRP [8,9]. The Epworth Sleepiness Scale is a validated questionnaire [10] with the ability to differentiate between normal sleepers and patients with sleep disorders, based on excessive daytime sleepiness. Patients who did not fulfil the above-mentioned criteria for OSA were excluded from the analysis.

The study was approved by the Local Ethics Committee of Constanța Clinical Pulmonology Hospital. All subjects gave their informed consent prior to their inclusion in the study.

### 2.2. Study Variables

A multidimensional analysis was performed for: (a) demographic and anthropometric relevant variables, (b) respiratory symptoms, (c) the existence of comorbidities, and (d) the specificities of obstructive sleep apnea related to smoking.

Smoking exposure was recorded by the pulmonologist in OSA patients who were divided into three different categories: current smokers (CS), those who had never smoked (NS), and ex-smokers (ES). An ES was defined as a person with a persistent smoking cessation of at least 12 months prior to study enrollment. Smoking exposure and dependence was assessed by the number of pack-years and the smoking dependence, using the Fagerström Test for Nicotine Dependence.

The demographic and anthropometric measures included age and gender distributions, weight, height, body mass index (BMI), and waist and neck circumferences.

OSA-related symptoms include gasping during sleep, awakening during sleep, sleepiness during the day, nocturia, and a morning headache. They were recorded qualitatively, except for excessive diurnal sleepiness, which was also measured quantitatively by the Epworth Sleepiness Scale.

OSA-related comorbidities were represented by cardiovascular disease, COPD, diabetes, ENT, and metabolic syndrome, and were included in the analysis.

The severity of OSA was classified based on the currently accepted international criteria [11], namely, OSA was mild if the AHI was between 5 and 15, moderate from 15 to 30, and severe if the AHI > 30.

The oxygen desaturation index (ODI) was extracted from the NRP. ODI was calculated, as previously described [12], as the number of times desaturations occurred over one hour, with ≥3% lasting at least 10 s. Other investigated oxygenation parameters were the lowest average SpO2 and the highest time spent below 90% of the oxygen saturation level during sleep (TS_SpO290).

All variables and parameters were recorded before the initiation of any specific treatment for OSA.

### 2.3. Statistical Analyses

Statistical analyses were performed with SPSS software (StatPlus: Mac, AnalystSoft Inc. Version v8, AnalystSoft Inc., Walnut, CA, USA). We performed a descriptive analysis of the study groups, calculating absolute frequencies and percentages for the demographical data, and for the sleep parameters. In the first step, all numerical data were checked for normality. After the preliminary normal distribution of data was rejected, the Kruskal–Wallis H test was applied to compare CS, ES, and NS. To assess the distribution of the categorical variables, the χ^2^ test was used. Due to the imbalance between groups regarding age and, especially, gender, χ^2^ was carried out on weighted data.

The correlation analysis of the smoking status indicators (the number of pack-years and the Fagerström score), on the one hand, and the severity of sleep apnea indicators (AHI < ODI and TS_SpO290), on the other, were performed using the Spearman test. Binary regression was used to assess the prevalence of the comorbidities according to the smoking status, and the results were adjusted for age and gender. An analysis of variance (ANOVA) was used to perform the univariate and the multivariate regressions. For all calculations, a *p*-value < 0.05 was considered statistically significant.

## 3. Results

**Figure 1:**
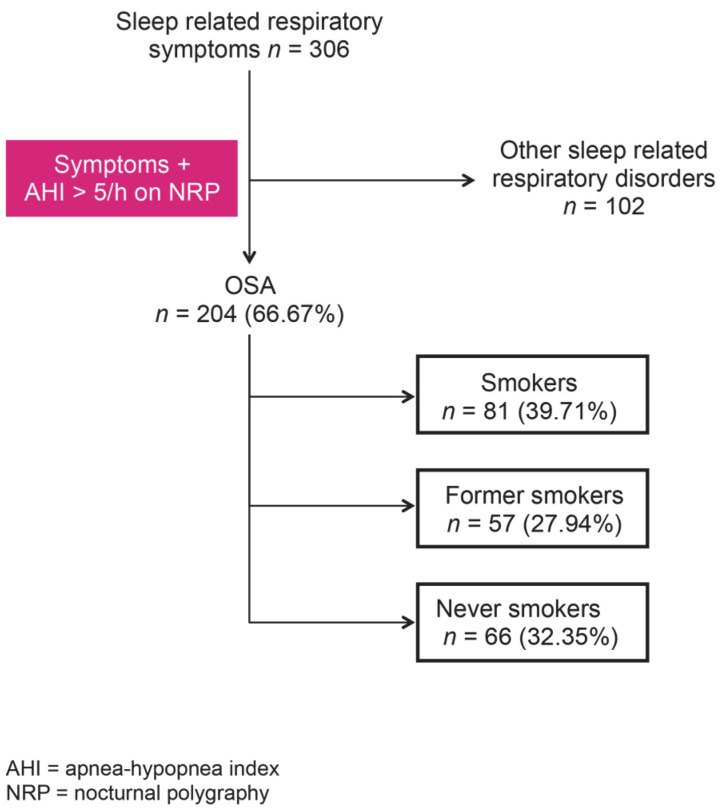
Selection of the study group.

Of the total of 306 patients with sleep respiratory symptoms, 204 fulfilled the criteria for OSA and were included in the following analysis. Among the OSA cases, 81 were CS, 66 were NS, and 57 were ES (Figure 1).

The average number of cigarettes quantified in pack-years was significantly higher in CS compared to ES (24.15 ± 10.6 SD versus 20.86 ± 10.45 SD; *p* = 0.04). On average, the duration of smoking abstinence among ES was 12.79 years ± 9.28 SD. Among ES, 45.61% quit smoking in the previous ten years, 15.8% between 11 and 15 years, 10.5% between 16 and 20 years, and 0.13% quit for more than 20 years. There was a direct correlation between the pack-years and the scale of tobacco dependence (Rho = 0.80, *p* < 0.001).

### 3.1. Demographic and Anthropometric Characteristics

The main characteristics of the study groups are presented in Table 1. The ES were the oldest, and the CS the youngest, with the age difference being statistically significant (H = 22.37, *p* < 0.001). When the comparison was restricted to CS and NS, both age and gender differences were maintained: H = 3.09, *p* = 0.04 and χ^2^ = 5.99, *p* = 0.01, respectively. The difference between ES and NS was marginally significant (H = 3.57, *p* = 0.06).

Age differences between CS and ES were also statistically significant (H = 22.37, *p* < 0.001). Similarly, when comparing CS to ES, the gender difference became statistically non-significant (χ^2^ = 0.002, *p* = 0.97). The percentage of ES was significantly higher in men than in women, compared to NS (52.15% vs. 12.12%, χ^2^ = 5.52, *p* = 0.02).

The BMI differences between CS and NS were not significant (H = 2.07, *p* = 0.14), while the waist and neck circumference differences among these two groups reached the threshold for statistical significance (H = 3.7, *p* = 0.050 and H = 4.1, *p* = 0.03, respectively). There was no significant difference in BMI, waist, and neck circumferences between ES and NS. The number of pack-years was also directly correlated to the neck (Rho = 0.19, *p* = 0.005) and waist circumferences (Rho = 0.18, *p* = 0.01).

We performed several regression models to reveal the significance of the number of pack-years, BMI, age, and gender as determinants of the waist and neck circumferences. The influence of the number of pack-years on these anthropometric characteristics is summarized in Table 2.

### 3.2. Analysis of SOSA-Related Symptoms

**Figure 2:**
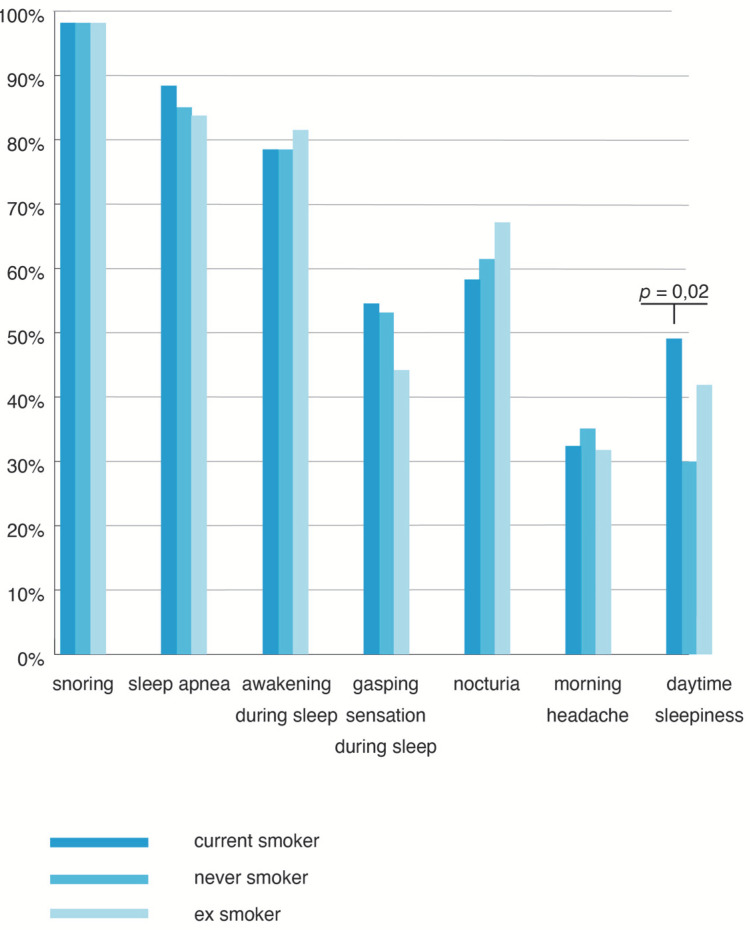
Prevalence of symptoms according to the smoking status. CS = current smokers; NS = never smoked; ES = ex-smokers.

All cases reported snoring. CS had the highest number of symptoms that suggested sleep apnea (gasping during sleep and sleepiness during the day), ES presented more frequent nocturia and awakening during sleep, and NS presented more morning headaches (Figure 2), but without statistical significance.

**Figure 3:**
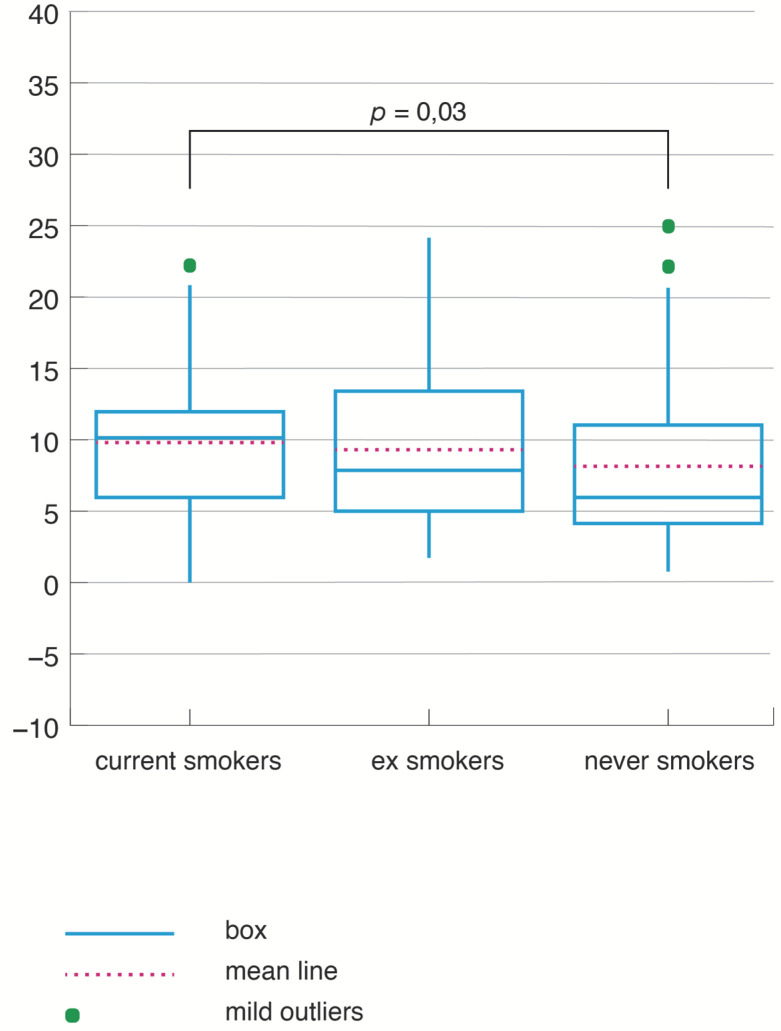
Epworth score according to smoking status.

When CS and NS were compared separately, the prevalence of daytime sleepiness became statistically significant (χ^2^ = 5.26, *p* = 0.02). In accordance with this result, the Epworth score was, also, significantly higher in CS compared to NS (average = 9.75 ± 5.1 SD in CS versus Average 8.26 ± 5.58 SD in NS; *p* = 0.03) (Figure 3).

The ES showed daytime sleepiness (χ^2^ = 1.89, *p* = 0.17) and the Epworth score average (9.33 ± 5.49 versus 8.26 ± 5.58, H = 1.62, *p* = 0.2) was similar to the NS.

### 3.3. Analysis of Comorbidities in SOSA

**Figure 4:**
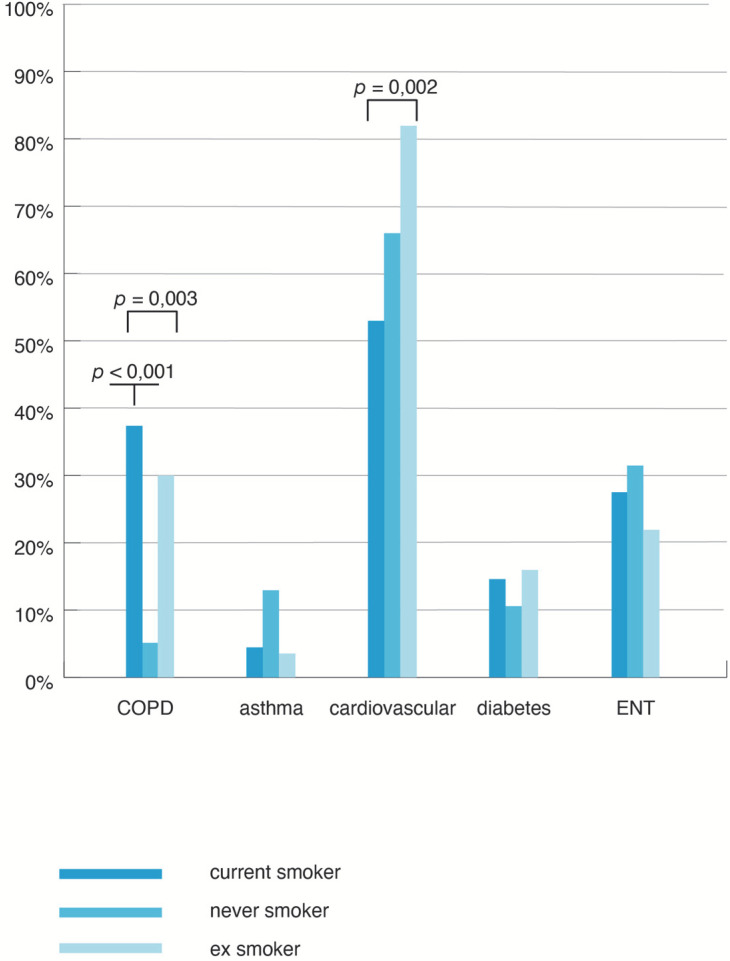
Point prevalence of comorbidities according to the smoking status. CS = current smokers; NS = never smoked; ES = ex-smokers.

The most prevalent comorbidities in OSA patients were cardiovascular diseases (67.65% of the total sample) followed by ear-nose-throat (ENT) disorders (29.41%), diabetes (15.20%), COPD (25.98%), and asthma (7.35%) (Figure 4).

Significant differences in the distribution of comorbidities among the three smoking groups were noticed only for cardiovascular diseases (χ^2^ = 11.99, *p* < 0.0001) and COPD (χ^2^ = 20.985, *p* < 0.0001). The cardiovascular diseases were more frequent in ES (84.21%) compared to CS (55.56%) and NS (68.18%). The prevalence was significantly higher in ES versus CS (χ^2^ = 19.9, *p* < 0.0001) and in ES and NS (χ^2^ = 49.9, *p* = 0.02). When adjusted for age and gender in a multivariate regression model, the smoking status was no longer statistically significant for the cardiovascular disease prevalence.

COPD was most frequently encountered in CS (38.27% of patients), followed by ES (31.57% of patients) and NS (1.51% of patients). The differences in the prevalence of COPD between CS and NS, and between ES and NS, were statistically significant (χ^2^ = 20.3, *p* < 0.001 and χ^2^ = 13.56, *p* = 0.0002, respectively). The relation between smoking and COPD was independent of the age variation in the multivariate regression model (*p* < 0.0001).

Asthma was more frequent in NS (13.63% versus 3.5% in ES and 4.993% in CS), but the difference was not statistically significant (χ^2^ = 5.75, *p* = 0.06). The percentage of patients with ENT disorders was 33.34% in NS, 24.56% in ES, and 29.63% in CS, which was also non-significant (χ^2^ = 1.14, *p* = 0.56).

### 3.4. Analysis of the NRP Study in SOSA

The mean values of the nocturnal respiratory polygraphy results are presented in Table 2. Overall, the NS showed better values of all the sleep parameters, reflecting a less severe OSA. Severe cases were more frequent in CS (80.24%), followed by ES (68.24%), and NS (56.06%) (Table 3).

The differences among the three groups were assessed with the Kruskal–Wallis test.

**Figure 5:**
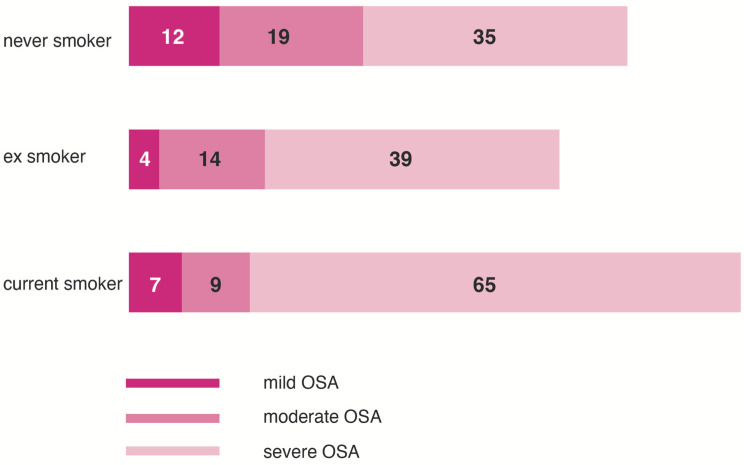
The severity of obstructive sleep apnea according to the smoking status. CS = current smokers; NS = never smoked; ES = ex-smokers; OSA = obstructive sleep apnea.

CS had significantly more frequent and severe forms of OSA than NS (χ^2^ = 5.3, *p* = 0.01) (Figure 5). The distribution of the AHI ranges of severity, between ES and CS, was not statistically significant (χ^2^ = 4.34, *p* = 0.22).

**Figure 6:**
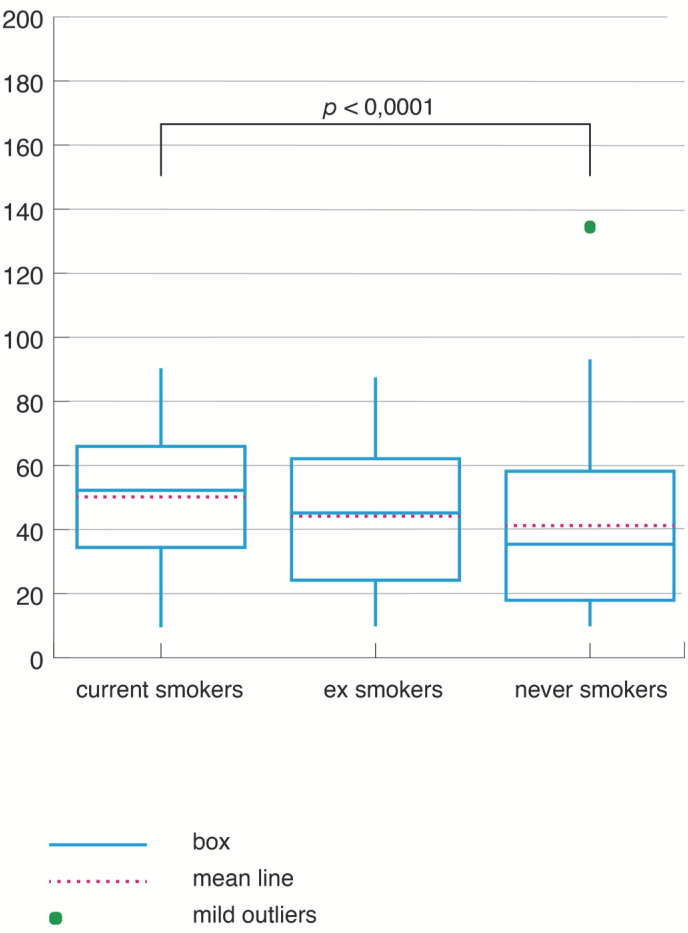
Apnea–hypopnea index according to smoking status.

As presented in Table 3, the values of the AHI were elevated in the CS group and were the lowest in the NS group. The difference was statistically significant when comparing CS to NS (H = 6.6, *p* < 0.0001) (Figure 6). The CS had a higher chance of severity of OSA, after adjusting for age, gender, and BMI (OR = 3.10, CI = 1.39–6.86, *p* = 0.005).

ES and NS had similar results for the AHI (H = 1.47, *p* = 0.22).

In the multivariate regression analysis, the direct relationship between the AHI and the number of pack-years was maintained, after adjusting for age, gender, and BMI. The OR for severe OSA was 1.03 (CI 95%: 1.014–1.06, *p* = 0.02) with the increasing number of pack-years.

**Figure 7:**
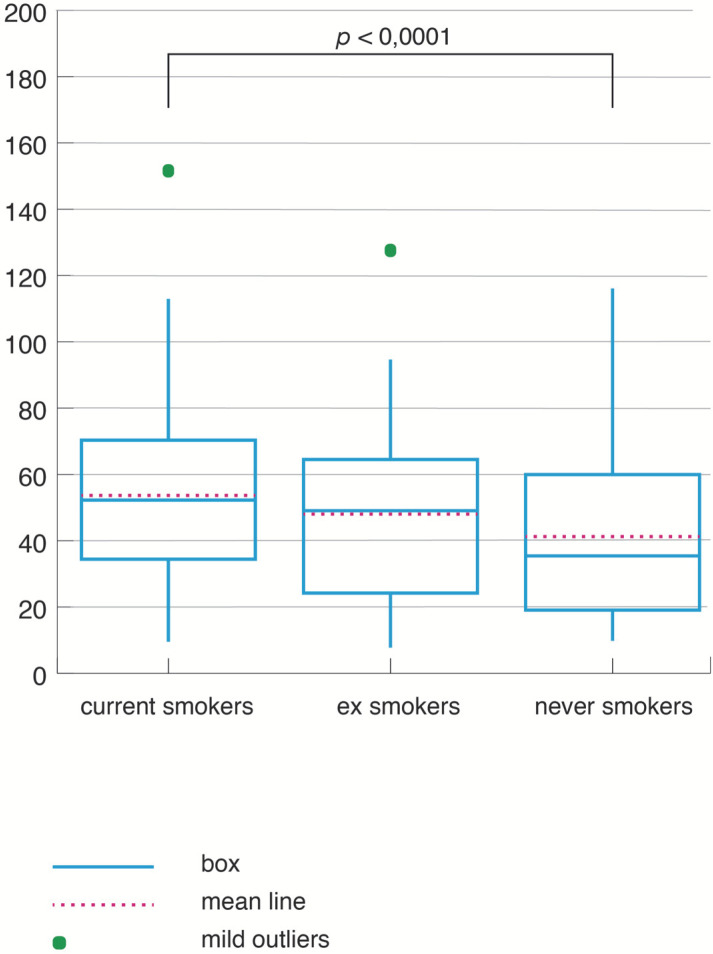
Oxyen desaturation index according to smoking status.

**Figure 8:**
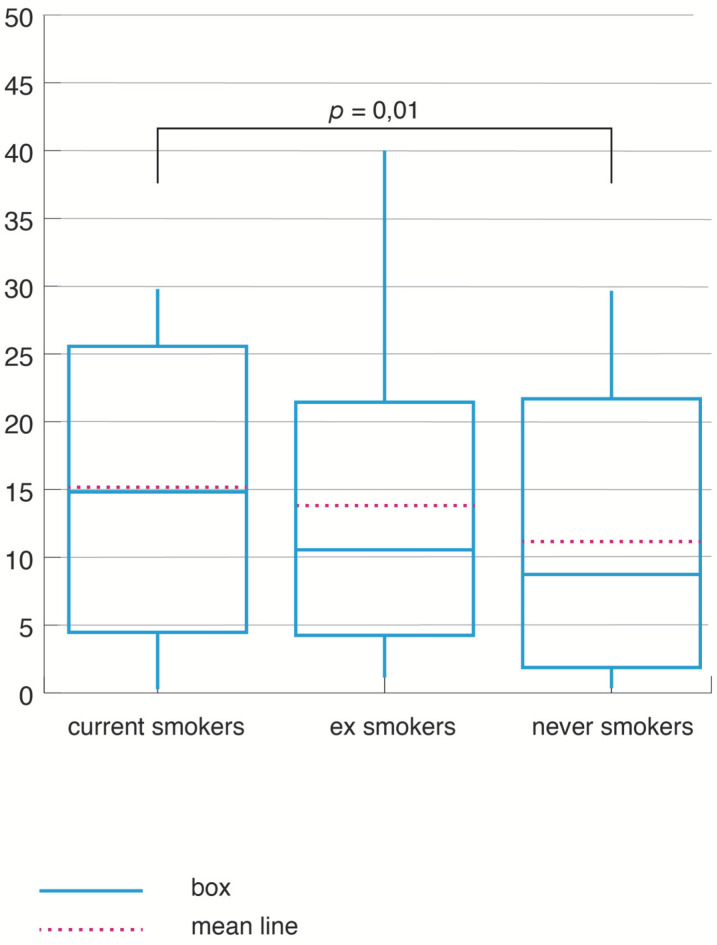
Time spent under 90% saturation in O_2_ during sleep according to smoking status.

The three groups of OSA patients also had distinct oxygenation parameters. CS had the lowest average SpO_2_, the highest ODI (Figure 7), and the highest time spent below 90% oxygen saturation level during sleep (TS_SpO_2_90) (Figure 8).

A significant difference was noticed in the comparison between CS and NS in TS_SpO_2_ (H = 5.40, *p* = 0.01) and ODI (H = 7.25, *p* < 0.0001). The differences related to the smoking status were not influenced by age and gender in this comparison: the relation between the smoking status and ODI maintained the statististical significance (coef = 10.26, CI = 1.44–19.08 *p* = 0.02 for ODI. In a similar way, the relation between the smoking status and TS_SpO_2_90 remained significant, after adjustment for age and gender (coef = 3.69, CI = 0.16–7.22 *p* = 0.04). When BMI was also introduced in the regression models, all other determinants lost their statistical significance.

Although the averages of ODI and TS_SpO_2_ were higher in ES than in NS, the level of statistical significance was not reached for either of these sleep parameters (H = 2.8, *p* = 0.09 and H = 2.57, *p* = 0.10, respectively).

The number of pack-years was directly correlated with the AHI (Rho = 0.157, *p* = 0.028), ODI (Rho = 0.15, *p* < 0.0001), and TS_SpO290 (Rho = 0.14, *p* < 0.0001) in CS (Figure 5). The Fagerström score was also directly related to the AHI (Rho = 0.15, *p* = 0.02), TS_SpO290 (Rho = 0.19, *p* = 0.006), and ODI (Rho = 0.17, *p* = 0.002) in CS.

The result of multivariate regression for the severe form of OSA (AHI > 30) is presented in Table 4.

The presence of COPD had a similar impact on the severity of OSA, independent of the smoking status (χ^2^ = 4.62, *p* = 0.32). In patients without COPD, smoking was related to a marginally higher chance for more severe OSA (χ^2^ = 8.10, *p* = 0.08).

When OSA severity was analyzed in patients with cardiovascular comorbidities, CS had the highest prevalence of severe OSA (84.44%), ES had an intermediate value (70.84%), and NS had the lowest prevalence (52.1%) (χ^2^ = 11.40, *p* = 0.02). In patients without any cardiovascular diseases, there were no significant differences in the severity of OSA in relation to the smoking status, although the highest prevalence was still recorded in the CS group (χ^2^ = 6.53, *p* = 0.10).

## 4. Discussion

To the best of our knowledge, this is a first-time study that highlights the SOSA phenotype in its defining traits. Even if the relationship between smoking and OSA is not a new finding, no previous study has analyzed the in-depth characterization of the SOSA phenotype. Our results show that SOSA patients have a higher waist and neck circumference, more frequent daytime sleepiness, a higher Epworth score, a higher prevalence of COPD, and worse sleep study parameters (higher AHI, ODI, and longer TS_SpO_2_). The number of pack-years and the Fagerström score are significantly correlated to all the severity markers of OSA. It is important to underline that the SOSA phenotype should be restricted to CS, while ES maintain, from the characteristics of SOSA, only a higher prevalence of COPD.

Like other epidemiological studies, the first distinctive feature of SOSA consists of a younger age. This should be considered as a specific trait which is also supported by other epidemiological studies [13]. It is generally accepted that the incidence of OSA increases with age, in both sexes, particularly after 60 years of age in women [14]. This is due to the changes in the area surrounding the pharynx (an increased deposition of fat and impaired muscular contraction) and the alteration of the genioglossus reflex, contributing to the pharyngeal collapsibility. Experimental data showed that these mechanisms interfere with smoking. The components of cigarette smoke act on the skeletal muscles, causing a loss of muscle mass, muscular fatigue, muscular capillary regression, and altered calcium handling [15]. Taken together, the consequences of smoking could accelerate the ageing process.

Most OSA patients are obese, but the BMI scores were not significantly different in the smokers and non-smokers of our sample. However, there was a direct relationship between the number of pack-years, central obesity, and the neck circumference, which seem to be other distinctive features of SOSA. This relationship was, notably, independent of BMI. Similar results were noticed in other cohorts in which current smokers exhibited a pattern of higher WC with increasing volumes of cigarettes smoked [16,17]. The strengths of these relationships are supported by a Mendelian randomization analysis of nearly 450,000 individuals, which showed that the genetic score for the waist circumference was positively associated with being a current smoker (OR = 1.33, CI = 1.21–1.46) [18]. In this analysis, the genetic influence was primarily driven by the SNPs clustering in the neuronal pathways, which is an argument for linking obesity with other risk behaviors (such as an unhealthy diet or sedentary behavior), which might prevail upon the direct effect of smoking on energy expenditure.

A direct relationship between smoking and waist circumference was found in other populational studies [16,19], but not in all [20]. An influence of gender on this association [13] (e.g., a greater influence in men than in women) was described, which might also be the case for our prevalent male sample. These particular findings seem relevant for the SOSA phenotype, because BMI scores and, more importantly, the central distribution of the adipose tissue, are considered the main risk factors for OSA [21]. Both waist and neck circumferences are of interest; one acts to decrease the longitudinal tracheal traction forces and pharyngeal wall tension, and the other reduces the transmural pressure in the pharynx [22]. A magnetic resonance imaging study of the upper airways and neck confirmed these mechanisms and showed a direct association with the severity of OSA [23].

On the other hand, OSA, per se, increases the risk of central obesity. The OSA patients who were obese had a distinguished pattern of circadian hormonal secretion (mainly of cortisol and testosterone) compared to obese patients [24]. A decreased level of testosterone in the evening [25], and chronic high levels of cortisol, contribute to the deposition of fat in the visceral region [26]. The combination of OSA pathophysiology with the higher levels of cortisol found even in healthy smokers in the morning [27] increases the risk of an unfavorable distribution of adipose tissue in the SOSA phenotype. The higher severity of OSA in AS could be a factor contributing to the relationship between smoking status and adiposity distribution.

In the multivariate analysis, the influence of the number of pack-years on the waist circumference was not dependent on age. This distinguishes SOSA from the trend of the waist circumference increasing with age, which has been observed in multiple cohorts [28]. On the contrary, gender attenuated the influence of the number of pack-years on the neck circumference.

In terms of symptoms, the main difference was noticed in daytime sleepiness and the Epworth score. In agreement with previous research [29], the Epworth scores are directly related to the AHI.

COPD and cardiovascular diseases had different distributions among OSA cases, according to the smoking status. In this study, COPD distinguished the CS group, while cardiovascular disease was mostly observed in the ES group. A reasonable explanation for the lower prevalence of cardiovascular comorbidities in CS is related to the early onset of OSA in younger adults. It is plausible that at least some of the former smokers quit smoking when their cardiovascular illnesses had already been diagnosed. On the other hand, from our data, active smoking counteracted the effect of aging on the decline of lung function. Smokers experienced an earlier decline in lung function, particularly in obstructive components, which was reflected in the diagnosis of COPD.

The coexistence of OSA and COPD defines the overlap syndrome. The prolonged hypoxemia associated with this syndrome decreases patients’ survival [30]. The negative effects of active smoking on the narrowing of the upper airways are enhanced by COPD. The reduction in the elastic recoil, related to lung emphysema, which decreases the tracheal traction forces, the systemic myopathy, the fatigue of the respiratory muscles, and the instability in breathing control, all contribute to the negative effects on the severity of OSA. Regarding these effects, the highest prevalence of OSA and COPD overlap syndrome in CS could contribute to the increased severity of OSA.

The severity of OSA was directly related to the active smoking status, with the highest AHI and lowest desaturation values at a younger age. In a larger study, a similar impact of smoking on the severity of OSA was shown, along with the fact that the smoking effect was more obvious in younger adults [19]. It is also important to underline that both the smoking status and the number of pack-years were correlated with the OSA severity in the multivariable regression. Moreover, the most severe OSA was independent of BMI scores, but it was still influenced by the number of pack-years, after adjusting for age and gender.

ES had an intermediate pattern of OSA severity revealed by the moderate alterations of the AHI, ODI, and TS_SPO_2_90, suggesting the positive influence of smoking abstinence. Due to the heterogeneity of the ES group, it is difficult to estimate the optimal duration of the abstinence period that is necessary to achieve a reduction in OSA severity. The direct correlation between the severity of OSA and the number of pack-years in smokers adds a quantitative effect of smoking to this relationship.

Even in non-OSA individuals, smoking is associated with poor sleep quality [31], increased desaturation [32], and diminished sleep continuity [27], which are well-known factors of influence of the AHI.

Another important factor is the effect of the smoking status on the severity of OSA in the presence of comorbidities. In patients with COPD, no differences in severity were noticed, but the number of non-smokers with COPD and OSA was very low (*n* = 6). On the other hand, the lack of a difference might be attributed to the general hypoxemic status induced by COPD, as well as sleep-related disorders in general, which are common in COPD; the shorter the sleep duration, the more frequent the impairment of sleep architecture in COPD patients, contributing to desaturation [33]. Additional data (e.g., the comparison of OSA and COPD, determined by occupational exposure in NS) are needed to bring up a conclusion.

In patients with cardiovascular disease and OSA, smoking represents an additional risk for severity, and this risk is the highest in the CS group.

There are several limitations of this study. Firstly, the number of patients (particularly in the NS group) was too low for conclusive remarks. However, data from other larger studies that included smoking analyses support our findings [28]. If there are differences (e.g., in the cardiovascular disease-associated comorbidity in the study by Shoa et al.), they might be the result of non-discriminating items between ES and CS. As we have shown, the differences between these two categories of patients are important, and the SOSA phenotype should be limited to CS patients. To confirm the results presented herein, an in-depth analysis of the smoking status in a large OSA cohort would be of benefit. Secondly, in this study, the OSA diagnosis was based on the nocturnal respiratory polygraphy. The diagnosis was performed following the recommendations of the international guidelines for clinical practice, but the RNP is not able to identify subtle modifications in the sleep architecture, as identified in other sleep studies in smokers. In this respect, adding the sleep architecture traits to the characteristics of the OSA-smoking phenotype would be of interest.

The number of women in the study was small, but this reflects the characteristics of the disease, which has a male-to-female ratio of between 3:1 and 5:1 in the general population [34]. There were only 13 AS in the female group, which did not allow for possible gender specificities.

The most important finding of the study is the identification of a specific phenotype in smoking patients with OSA. Even if it is not a very large study, the results are supported by other epidemiological data and by plausible pathophysiology underlying the specific effects of smoking in OSA [15,16]. The recognition and the better characterization of what we propose as the SOSA phenotype not only has a theoretical value, but it also has an impact on patients’ clinical management and opens the field for research dedicated to this subgroup of patients. It is generally assumed that both smoking cessation [12] and weight reduction [35,36] are beneficial to improve nocturnal desaturation, but The National Lung and Heart Institute does not recommend initiating them simultaneously [32]. There is no general indication regarding which of these two interventions should come first, but the European Respiratory Society Task Force underlines that “patients with respiratory diseases have a greater and more urgent need to stop smoking than the average smoker” [32]. Even if not specifically mentioned, the OSA smokers should be included in the category of respiratory patients in need of an urgent procedure of smoking cessation. On the other hand, considering the benefits from a reduction in adipose tissue on the breath mechanics and on systemic inflammation, The American Thoracic Society strongly recommends “participation in a comprehensive lifestyle intervention program that includes a reduced-calorie diet, exercise/increased physical activity, and behavioral counseling rather than no program” [33]. A multidisciplinary intervention is of particular interest after the initiation of CPAP therapy, or when a smoking cessation program is initiated, as both of these circumstances potentially promote weight gain. As there are no current clinical traits to evaluate what the most efficient intervention in OSA smokers is, experts emphasize that the decision should be tailored according to patients’ choices [37,38]. Future research might indicate whether the general effects of lifestyle interventions (e.g., the mitigation of the arterial stiffness by aerobic exercise and no negative effects of weight loss programs on smoking behavior) could also be applied to OSA patients, which may provide a better outcome to patients.

Lung function should be carefully assessed in SOSA patients to promptly identify the decline in flow during the first second of forced expiration (FEV1) in relation to the occurrence of COPD, and to initiate the appropriate therapeutic measures. This association also has another clinical implication, which involves actively investigating SOSA in COPD patients, as well as adding specific OSA therapies to the clinical management of COPD, whenever necessary.

## 5. Conclusions

We have shown that the SOSA phenotype is characterized by the following parameters: male predominance, an earlier onset (during the fifth, and the beginning of the sixth, decade), abdominal fat deposition, a higher prevalence of COPD, and a higher severity of COPD. The severity of OSA correlates with the number of pack-years. The SOSA phenotype should be restricted to active smokers, as former smokers might have a different pathological profile. Smoking cessation and bodyweight reduction are the two key lifestyle changes to be recommended in SOSA in comprehensive clinical management to achieve sustainable results.

## Figures and Tables

**Table 1:** Demographics and anthropometric characteristics of OSA patients.

	CS (*n* = 81)	NS (*n* = 66)	ES (*n* = 57)	Total	*p*
Age (years)					
Average	50.06 ± 9.23	52.26 ± 12.47	57.47 ± 8.52	52.84 ± 10.61	0.00006
Median	52	56	59	56	
Gender (M/F)	68/13	44/22	48/9	160/44	0.018
% Men	83.95%	66.67%	84.21%	78.43%	
BMI (kg/m^2^)					
Average	36.17 ± 6.97	34.35 ± 6,82	34.84 ± 6.12	35.21 ± 6.71	0.31
Median	35	34	34	34	
Waist circumference (cm)					
Average	113.34 ± 10.59	109.53 ± 10.99	111.93 ± 8.58	111.71 ± 10.29	0.13
Median	112	108	111	110	
Neck circumference (cm)					
Average	44.44 ± 3.39	43.26 ± 3.78	44.23 ± 3.03	44 ± 3.45	0.07
Median	44	42	44	44	

Legend: CS = current smokers; NS = never smoked; ES = ex-smokers; BMI = body mass index.

**Table 2:** Relation between anthropometric characteristics and number of pack-years.

	Number of Pack-Years			
Anthropometric Characteristics	Coefficient	SE	CI	*p*
Waist circumference	0.08 *	0.04	0.159–2.068	0.04
	0.12 **	0.05	0.02–0.22	0.04
	0.09 ***	0.05	−0.007–0.20	0.06
Neck circumference	0.03 *	0.02	0.004–0.065	0.02
	0.02 **	0.016	−0.01–0.05	0.22
	0.019 ***	0.02	−0.01–0.05	0.23

SE = standard error; CI = confidence interval; * data adjusted for BMI, ** data adjusted for age, *** data adjusted for gender.

**Table 3:** Parameters of the sleep study according to the smoking status.

Sleep Characteristics	CS	NS	ES	Total	*p*
AHI					
Average ± SD	50.44 ± 22.17	41.48 ± 27.19	44.58 ± 21.38	45.91 ± 23.91	0.03
Median	53	35	45	45	
Average SpO_2_					
Average ± SD	90.81 ± 3.97	91.52 ± 4.69	91.44 ± 3.12	91.22 ± 4.01	0.12
Median	92	93	92	92	
Minimal SPO_2_					
Average ± SD	72.84 ± 8.23	73.32 ± 7.67	72.72 ± 7.01	72.96 ± 7.69	0.83
Median	72	73	72	72.5	
TS_SpO_2_90					
Average ± SD	15.07 ± 10.81	11.27 ± 10.15	13.71 ± 10.04	13.47 ± 10.46	0.057
Median	15	8.5	11	11	
ODI					
Average ± SD	53.43 ± 27.59	41.5 ± 26.50	47.96 ± 25.38	48.04 ± 26.98	0.02
Median	52	34.5	49	46	

CS = current smokers; NS = never smoked; ES = ex-smokers; AHI = apnea–hypopnea index; SpO_2_ = peripheral saturation in oxygen; TS_SpO_2_90 = time spent below 90% of the oxygen saturation level during sleep; ODI = oxygen desaturation index; SD = standard deviation.

**Table 4:** Determinants of the severity of OSA.

	Severity of OSA			
	Coefficient	SE	CI	*p*
Age	−0.02	0.02	0.95–1.01	0.33
Gender	−0.03	0.45	0.40–2.35	0.95
Waist circumference	0.13	0.024	1.09–1.20	<0.001
Number of pack years	1.03	0.01	1.003–1.06	0.02

SE = standard error; CI = confidence interval.

## Data Availability

Data are available from the first author upon reasonable request.
